# Relapse vs. Re-Infection

**Published:** 1919-03

**Authors:** Robert W. Barton

**Affiliations:** Marion, Arkansas


### A DISTINCTION WITH A DIFFERENCE.

### Relapse Vs. Re-Infection.

ROBERT W. BARTON, M. D., MARION, ARKANSAS.

Editor Texas Medical Journal.

Dear Editor: The treatment of flu, or influenza; its liability to the complication of pneumonia; its tendency to relapse on short duration; of immunity after an attack, are now of prime interest to the medical profession.

In the treatment of influenza I have had an experience that is gratifying, having never had a death from uncomplicated influenza. In fifteen cases complicated with pneumonia I have had thirteen cases recover. My treatment of the disease was confined to practically the same remedies in all of the 106 uncomplicated cases: (1.) Confinement to bed during the disease and for several days after disappearance of fever. (2.) Strictly a buttermilk diet with lemonade ad libitum while fever existed. (3.) A purge of cal-co, salts or mineral oil as indicated, quinine 5 grains from three to four times a day alternated with not less than five grains of uritone three times daily, a mentholated salve to apply in nostrils and rub around throat and chest.

The above treatment seemed to bring rapid improvement in all cases of uncomplicated influenza.

When pneumonia developed, in addition to the above treatment I at once applied a cotton-wool jacket, viz.: a surgical cotton lined jacket, completely covering the whole chest. To control excessive fever and very rapid pulse I gave veratrum Norwoods Tr. 3 to 5 drops; Tr. aconite root 1 to 2 drops; bicarbonate soda 2 to 3 grains every four to six hours as indicated, and in all but two cases of the fifteen pneumonias I administered every twenty-four hours until three or four doses were given, Pneumo-Sero-Bacterins (mixed) a. b. c. d.

The most interesting experience in which I am of the opinion that I am permitted to make a discovery of great value and which I hasten through your columns to give to the profession, and through them to humanity, is connected with my personal experiences of a dangerous attack of this disease. In August, 1918, I took a treatment of four doses at weekly intervals of the Influenza Bacterin (plain) to guard me against a marked tendency to cold, sometimes followed by bronchial asthma. I was constantly exposed to the epidemic to the point of exhaustion and had to go to the hospital twice during the epidemic from overwork with marked nervous prostration, each time for a week, but did not contract the disease until December 27th. On December 28th I went to a hospital and was dangerously sick from influenza for twelve days. On January 9th, after twelve days' confinement to the hospital in Memphis, I recuperated for a week in a hotel at Jonesboro, Ark. On the 16th of January I returned to my office but found recovery very slow —a pleurodynia and a decided weakness with a constant suffering in the joints, particularly in the knees, convinced me of the seriousness of this disease. The neuralgia of the pleura in my right chest was temporarily relieved by the application of chloroform on paper applied to the affected art. My experience in convalescence convinced me that the recurrence of attacks of this disease that have led to the professional opinion that immunity after an attack was very short, often not over ten days, is a mistake; for the reason that what is really a relapse in the same attack is confused with a new infection. I reasoned from my own experience that my slow recovery, the suffering in the joints, the pleurodynia with its great pain, was proof that I was still infected and that the return of active febrile symptoms, with or without pneumonia, were liable at any time to recur from over exertion, from getting wet or exposure to drafts, chilling the body, rekindling into activity, a condition that had become sub-acute or chronic, with the result that a relapse would occur.

Believing this, I reasoned that the Influenza-Sero-Bacterins mixed should kill this systemic infection of convalescence and destroy the latent germs, or it may be, the toxines left from the affect of the germs of the disease.

I have made the experiment on myself. The first dose of the serum within six hours made an improvement and within twenty-four hours a decided improvement of all symptoms. After the second dose of serum all "flu" symptoms had disappeared, even to excessive excretions from mucous membranes of nose and throat.

I have deduced from the above experience that the prompt administration of the Influenza-Sero-Bacterins mixed after fever has subsided in an influenza attack will abort the long convalescence, will cure or make well the one attacked, and prevent the long convalesence in which the constant liability to a fatal relapse exists.

I earnestly invite the attention of the profession to my successful effort to promptly cure your influenza patients by eradication of the germs of the disease, left latent in various degrees of infection in all cases of influenza, thereby preventing relapse, probably pneumonia with fatal results; and even the saving of time and labor to the nation by such a course is incalculable.

If my views are found sound by the profession I will count my personal suffering, loss of time and expense, to say nothing of the risk of life, all gain.

